# Micro-Solvated DMABN: Excited State Quantum Dynamics and Dual Fluorescence Spectra

**DOI:** 10.3390/molecules26237247

**Published:** 2021-11-29

**Authors:** Sandra Gómez, Esra N. Soysal, Graham A. Worth

**Affiliations:** Department of Chemistry, University College London, 20 Gordon Street, London WC1H 0AJ, UK; e.n.soysal@sms.ed.ac.uk

**Keywords:** dual fluorescence, excited states, nonadiabatic dynamics

## Abstract

In this work, we report a complete analysis by theoretical and spectroscopic methods of the short-time behaviour of 4-(dimethylamino)benzonitrile (DMABN) in the gas phase as well as in cyclohexane, tetrahydrofuran, acetonitrile, and water solution, after excitation to the L${}_{a}$ state. The spectroscopic properties of DMABN were investigated experimentally using UV absorption and fluorescence emission spectroscopy. The computational study was developed at different electronic structure levels and using the Polarisable Continuum Model (PCM) and explicit solvent molecules to reproduce the solvent environment. Additionally, excited state quantum dynamics simulations in the diabatic picture using the direct dynamics variational multiconfigurational Gaussian (DD-vMCG) method were performed, the largest quantum dynamics “on-the-fly” simulations performed with this method until now. The comparison with fully converged multilayer multiconfigurational time-dependent Hartree (ML-MCTDH) dynamics on parametrised linear vibronic coupling (LVC) potentials show very similar population decays and evolution of the nuclear wavepacket. The ring C=C stretching and three methyl tilting modes are identified as the responsible motions for the internal conversion from the L${}_{a}$ to the L${}_{b}$ states. No major differences are observed in the ultrafast initial decay in different solvents, but we show that this effect depends strongly on the level of electronic structure used.

## 1. Introduction

The anomalous spectroscopic characteristics of the donor–acceptor compound 4-(dimethylamino)benzonitrile (DMABN) were first reported in 1962 [1] by the group of E. Lippert, who found that the molecule showed two fluorescence bands in high polarity solvents, while the parent compound aminobenzonitrile (ABN) lacked such an effect. Little agreement has been found since then, starting by the acronym used: the molecule is known also as NNDMPCA [2], DAB-I [3] and p-cyano-dimethylaniline (CDMA) [4], although DMABN has become the established acronym.

The state reversal model postulated in 1962 by Lippert et al. [5] only considered the diabatic states L${}_{b}$ and L${}_{a}$ that correspond to the first and second excited states at the ground state optimised geometry. “L” points out the local excited character of the states, while a and b match the irreducible representation to which the state belongs to within the C${}_{2}$ point group.The original notation was coined by Platt [6] and referred to free atom term symbols, acknowledging that the state character corresponds to electrons coming from and going to π orbitals. This theory assumed that, upon excitation to the first absorption band, both L${}_{b}$ and L${}_{a}$ are excited and ultrafast internal conversion from L${}_{a}$ and vibrational relaxation on the $L_{b}$ state take place until the normal emission occurs from the L${}_{b}$ minimum. The anomalous emission in the presence of the solvent is then attributed to a solvent reorganization that stabilises the L${}_{a}$ state minimum with respect to the L${}_{b}$ state. The state reversal model was soon discarded when the fluorescence anisotropy was remeasured and found to be positive in both emission bands, while Lippart had found the bands to be perpendicular to each other. Additionally, the Stokes shift was too large (0.8 eV) to be solely attributed to a solvent effect. Other plausible explanations that followed included proton transfer and hydrogen bonding [7], ruled out due to the dual fluorescence also present in aprotic polar solvents, the formation of excimers formed by dimerization [2] and the formation of exciplexes with the solvent [8].

The theory assigning the anomalous fluorescence to a different excited state conformation was first introduced in 1973 by Rotkiewicz, Grabowski and co-workers [3]. They concluded that the normal emission band had to be produced by a coplanar conformation, while the anomalous band corresponded to a 90${}^{\circ }$ twisted conformation produced by a charge transfer from the methylene group to the $\pi_{y}$ orbital in the cyano group. They named the structure torsional (or twisted) internal charge transfer (TICT).

Among the other theories that attribute the anomalous band to specific excited state conformations are (i) a pseudo Jahn-Teller coupling between the L${}_{b}$ and L${}_{a}$ states involving an inversion of the amino group [9,10], sometimes also called the wICT model (wagging-ICT), (ii) a rehybridized ICT (RICT) state possessing a bent cyano group [11,12], (iii) a partially twisted geometry (pTICT) [13], (iv) a planar intramolecular charge transfer state (PICT) [14,15,16] and (v) the involvement of a πσ* state [17]. Every hypothesis regarding the emission mechanism in DMABN that was postulated before 2003 is well documented in the review by Grabowski et al. [18].

The TICT state theory, that claims that an internal rotation charge transfer state is responsible for the anomalous emission is the most accepted theory and has been supported by superjet cluster measurements [19], fragment orbital interaction and excited state energy decomposition analysis [20], by measurements in DMABN derivatives, the torsion of which was impeded sterically [21], by excitation to the red edge band [22] and by solvatochromic analysis of absorption and emission data [23].

From the theoretical point of view, high level CASPT2 simulations have been performed on DMABN in the gas phase, acetonitrile [24], water [25,26] and tetrahydrofuran [26]. All show that the TICT minimum is more favourable than the PICT minimum and that the influence of the πσ* state can be ruled out. This conclusion, however, seems to change with the number of states used in the state average CASSCF, since studies by the Robb group showed that the first step after irradiation is relaxation to the PICT minimum on the S${}_{2}$ state, followed by internal conversion to the S${}_{1}$ and equilibration between the LE and TICT states [27]. They found that the S${}_{2}$/S${}_{1}$ intersection seam may be accessed at many torsion angles. The ICT state has also been characterised by CC2 and CCSD methods [28] including excited state frequencies, finding that that state has a minimum geometry belonging to the C${}_{s}$ point group instead of the C${}_{2v}$. Recently, Kretz et al. have published the success of TDDFT range-separated functionals such as CAM-B3LYP and OT-RSH in describing the CT and LE excited states of DMABN [29], whereas PBE and B3LYP give an incorrect result.

To include the effect of the solvent, the algebraic diagrammatic construction method (ADC2) has been used to describe DMABN in benzene, DMSO and water together with ASEC and COSMO solvation models [30], showing that the topology of the PES along the torsion is very similar for each solvent. A QM/MM approach using TDDFT and AMOEBA has also been used to describe the equilibration between the LE and TICT states of DMABN in acetonitrile on the S${}_{1}$ surface using Born-Oppenheimer molecular dynamics (BOMD) [31].

Regarding nonadiabatic dynamic studies, DMABN in the gas phase has been used as a test system for the surface hopping program JADE [32] using TDDFT (CAM-B3LYP) for the electronic structure and 50 trajectories generated from a Wigner distribution started in the S${}_{2}$ adiabatic state. Ultrafast internal conversion to the S${}_{1}$ state was observed in less than 10 femtoseconds. Kochman et al. published in the same year very similar results on the gas phase system, using 24 surface hopping trajectories generated from a Wigner distribution and ADC2 for the electronic structure [33]. In a later publication in 2020, Kochman and Durbeej improved the previous setup using QM/MM on DMABN in a water nanodroplet, and observed for the first time the formation of the TICT state at around 600 fs. Very recently, they have applied the CC2 method to compute the transient absorption spectra of DMABN in acetonitrile [34].

The group of Subotnik some years later studied this ultrafast internal conversion (200 surface hopping trajectories generated from ground state molecular dynamics simulations, TDDFT/wB97X) and observed some differences between the rate of decay of the adiabatic and the diabatic states in the gas phase and in a micro-solvated environment (DMABN + one acetonitrile molecule), but concluded that nuclear wavefunction simulations were needed to yield accurate diabatic decay rates and to weight the effect of the geometric phase on this system [35]. Finally, Curchod et al. used a frozen Gaussian method to study the full quantum nuclear dynamics in this system and applied Ab Initio Multiple Spawning (AIMS) with TDDFT and the ωPBE functional to the gas phase single molecule [36]. Their 21 independent trajectories generated from a Wigner distribution (that increased to 186 due to the spawning) confirmed that the dimethyl torsion is not involved in the early S${}_{2}$/S${}_{1}$ transfer and that only the phenol C=C ring bonds showed sizeable variations during this short time. In a later publication, Ibele and Curchod pointed out that, during this fast transfer, different points of the intersection seam were visited and that one would expect a branching of the nuclear wavepacket without a posterior recombination [37].

In this study, we investigate the ultrafast relaxation of DMABN using quantum dynamics methods in full dimensionality and in explicit micro-solvated environments (water, acetonitrile and tetrahidrofurane). Our detailed study, using CAS and TDDFT methods for the quantum chemistry and a plethora of quantum dynamics methods (DD-vMCG, MCTDH, ML-MCTDH), as well as absorption and emission spectroscopies, aims to contribute to the long discussion about the deactivation mechanisms in DMABN after light irradiation.

## 2. Methods

### 2.1. Experimental Section

For the experimental part of this work, DMABN was purchased from Aldrich (Purity 98%, MW: 146.19 g/mol, mp: 72–75 ${}^{\circ }$C, bp: 318 ${}^{\circ }$C) and used without further purification. Solutions of DMABN in cyclohexane, tetrahydrofuran, acetonitrile, and water were prepared at room temperature. Concentrations of these solutions within the range of $10^{-6}$ to $10^{-3}$ M were measured. All solvents were commercially obtained from several chemical companies and used without further purification. The solvents did not show any traces of impurities in absorption and fluorescence measurements. In Table 1, a list of the solvents used in this work is given along with their physical characteristics where ϵ is the dielectric constant and n is the refractive index of the solvent.

The absorption measurements were made using a Perkin Elmer UV/VIS Lambda 365 spectrophotometer. The fluorescence emission spectra were recorded with a Horiba Jobin-Yvon Fluorolog-3 spectrofluorometer using a Xenon lamp as a source of beam excitation at 290 nm (4.27 eV). Fluorescence spectra were collected in the signal/reference (S/R) mode to correct for the changes in the lamp output intensity. Samples for absorption and emission measurements were prepared in a 1.0 cm path length of quartz cuvettes. Solvent blanks were subtracted from both the absorption and fluorescence emission spectra before the analysis.

### 2.2. Electronic Structure

For the static electronic structure calculations, the highest level of theory used was SA(4)-MS-CAS(12,11)PT2 using the cc-pVDZ Dunning basis set in the program OpenMolcas [38]. A picture of the active space used can be found in the Supplementary Materials (Figure S1) and can be decomposed as two electrons for the NH${}_{2}$ lone pair, six electrons in six orbitals for the benzene ring and four electrons in four orbitals for the π system of the cyano group. No imaginary level shift and no IPEA shift were used. Computations at the SA(4)-MS-CAS(6,5)PT2/cc-pVDZ were also performed, since for parametrisation of the potential energy surfaces around the Frank-Condon region the larger active space was found to be unstable (not providing balanced CASSCF reference weights for every electronic state considered in the state average) for some of the geometries. These energies were compared to EOM-CCSD with the def2-SVP basis and TDA-TDDFT using the wB97X-D3 functional with the cc-pVDZ basis set using the program Q-Chem [39]. Vertical energies were calculated for the optimised ground state geometry calculated with MP2/cc-pVDZ for the CAS and EOM cases and for a DFT optimised geometry with the same functionals for the TDDFT calculations.

The solvent effects were tackled using the polarisable continuum model (PCM) [40,41]. For the CAS vertical energy computations, the dielectric version was used and the slow component of the solvent response was kept frozen i.e., the charges computed from the equilibrium calculation of the ground state were used. The fast part of the solvent response was instead computed self-consistently for the state of interest, performing a state specific calculation. For the TDDFT energies and excited state optimisations, the conductor version of PCM (C-PCM) was used, adding two explicit solvent molecules for every solvent studied except for cyclohexane. The number of explicit solvent molecules was tested for the water/DMABN cluster and we found that DMABN with one solvent molecule at each polar end was the minimal system necessary to describe solvent effects. The cavity was constructed using van der Waal atomic radii multiplied by 1.2, except for the hydrogen atom where a value of 1.1 was used, which is the default in Q-Chem [42]. The surface was discretised using the switching Gaussian method (SWIG). For geometry optimisations, the static dielectric constant was used in the C-PCM calculations, considering the solvent in equilibrium with the solute, whereas a perturbative single state (ptSS) and linear response (ptLR) corrections applied to the zeroth order solvent polarised electronic wavefunction [43], was the method used to correct excited state energies for the model Hamiltonians used in the quantum dynamics simulations described below.

### 2.3. LVC Parametrised Potentials

Model Hamiltonians were constructed using the linear vibronic coupling (LVC) model [44,45]. In this model, the ground state potential energy surface is constructed as the sum of harmonic oscillators for each normal mode in mass-frequency scaled coordinates, while the excited state surfaces *n* are a vertical projection of the ground state surfaces shifted along the modes *i* that have a non-zero gradient, $\kappa_{i}^{\left(n\right)}$. The model uses a diabatic ansatz for the electronic states and the interstate coupling is provided by linear terms with parameters $\lambda_{i}^{\left(m,n\right)}$. The potential operator matrix is thus

(1) $$\begin{array}{cc} V_{0} & =\sum_{i}\frac{\hbar \omega_{i}}{2}q_{i}^{2} \end{array}$$

(2) $$\begin{array}{cc} W_{nn} & =E_{n}+\sum_{i}\kappa_{i}^{\left(n\right)}q_{i} \end{array}$$

(3) $$\begin{array}{cc} W_{mn} & =\sum_{i}\lambda_{i}^{\left(m,n\right)}q_{i} \end{array}$$

Here the $E_{n}$ are the vertical excitation energies, while the $\kappa_{i}^{\left(n\right)}$ and $\lambda_{i}^{\left(m,n\right)}$ are termed intrastate and interstate vibronic coupling constants [44]. The parameters are obtained from quantum chemistry calculations at the Franck-Condon point. Taking the gauge for the diabatic basis by setting it equal to the adiabatic basis at the Franck-Condon point, the diabatic interstate nonadiabatic coupling at the optimised geometry is defined as the derivative of the electronic Hamiltonian with respect to the normal mode displacements, $\langle \Phi_{m}|\frac{\partial H_{\mathrm{el}}}{\partial q_{i}}|\Phi_{n}\rangle$, with a magnitude that is related to the nonadiabatic force matrix element that can be obtained directly from the electronic structure calculations.

### 2.4. Quantum Dynamics

DD-vMCG is a frozen Gaussian nuclear dynamics method that shares the wavefunction *ansatz* with the full multiple spawning method of Curchod and Martínez [37,46] and the coupled coherent method of Shalashilin [47], but both these methods use classical trajectories to propagate the Gaussian centres, while in vMCG the Gaussian basis functions follow variationally determined trajectories to keep the basis set optimally small. The method has been describe extensively in the literature [48,49,50] and will only be outlined here.

In the DD-vMCG method, we express the nuclear wavefunction as a linear combination of *n* frozen Gaussians $g_{j}$

(4) $$\Psi \left(q,t\right)=\sum_{j=1}^{n}\sum_{s=1}^{n_{s}}A_{js}g_{j}\left(q,t\right)|s\rangle .$$

where |s〉 is the electronic state vector.

The set of parameters Λ of the Gaussians $g_{j}$ and the individual amplitudes A${}_{j}$ evolve under the Dirac-Frenkel variational principle [51,52] following equations of motion that can be written in a very compact notation as

(5) $$i\dot{\Lambda }=C^{-1}Y$$

and

(6) $$i{\dot{A}}_{js}=\sum_{k,l=1}^{n}\sum_{t=1}^{n_{s}}S_{jk}^{-1}\langle sg_{k}\;|\;H\;|\;g_{l}t\rangle A_{lt}-\sum_{k,l=1}^{n}iS_{jk}^{-1}\langle g_{k}|\frac{\partial }{\partial t}g_{l}\rangle A_{ls}$$

The DD-vMCG method takes advantage of previously calculated electronic structure points stored in a database to construct the full potential energy surfaces [50]. Initially, a calculation including energies, gradients, nonadiabatic couplings and Hessians at the Frank-Condon point is carried out with the electronic structure method of choice. As the dynamics progress, the database is filled with electronic structure points transformed to the diabatic picture using the propagation diabatisation method [53]. New electronic structure points are calculated only if a nuclear structure is significantly different from any of the geometries stored in the database, otherwise Shephard interpolation [54] between the diabatic energies is performed. Propagating in the diabatic picture circumvents problems related to geometrical phase effects that can be found in the adiabatic picture.

The on-the-fly DD-vMCG dynamics were carried out in the Quantics package [55,56]. Propagations start by projecting one single Gaussian that corresponds to the vibrational eigenstate of the electronic ground state at 0 Kelvin onto the $L_{a}$ excited state. The other Gaussians in the nuclear basis have zero amplitude and are displaced in momentum space, while sharing the coordinate centre. This is done to alleviate the computational cost at the first time step when the database of ab initio points is created, since we need to calculate gradients and Hessians of every electronic state involved. The variational nature of the equations of motion means the method is insensitive to the initial conditions of unpopulated Gaussians and there is no sampling of Gaussian weights as required in trajectory methods. The initially zero weighted Gaussians will evolve under equations of motion governed by the Dirac-Frenkel variational principle to accommodate the shape of the wavepacket during the dynamics.

Eight Gaussian wavepackets were used for the simulations in the gas phase, that include all 57 nuclear degrees of freedom for DMABN, and 75 nuclear degrees of freedom for the microcluster formed by DMABN and two water molecules. For the reduced dimensionality, four dimensional calculations (denoted 4D-DD), 40 Gaussian wavepackets were used in the case of the single DMABN molecule, whereas 20 Gaussians were used for the more expensive water cluster. To demonstrate the convergence of the calculations, simulations on the complete DMABN molecule in the gas phase with different numbers of electronic states and different nuclear basis sizes (up to 100 Gaussians using a partition method, i.e., lower dimensional Gaussians forming a direct product basis [57]) can be found in the Supplementary Materials (Figures S5 and S6).

For the DD-vMCG dynamics, the electronic structure method used was TDA-TDDFT with the functional wB97X-D3 and the Dunning cc-pVDZ basis set, as implemented in Q-Chem5.3. Analytical derivative couplings and gradients were used. To benchmark the direct dynamics, accurate grid-based quantum dynamics were used to solve the time-dependent Schrödinger equation. For these, the multilayer multiconfigurational time-dependent Hartree (ML-MCTDH) method was chosen as the only general method available for the propagation of wavepackets made here which had up to 135 degrees of freedom, as required for micro-solvated DMABN with tetrahydrofuran.

In the MCTDH method [58], the nuclear wavefunction is expressed as a linear combination of multidimensional single particle functions (SPFs) φ in the *k* degree of freedom (or combination of degrees of freedom)

(7) $$\Psi \left(q,t\right)=\sum_{j_{1}=1}^{n_{1}}\ldots \sum_{j_{p}=1}^{n_{p}}\sum_{s=1}^{n_{s}}A_{j_{1}\;,\cdots ,\;j_{p},s}\left(t\right)\prod_{k=1}^{p}\varphi_{j_{k}}^{\left(k\right)}\left(q_{k},t\right)|s\rangle .$$

In ML-MCTDH [59], these SPFs are in turn expressed as a linear combination of lower-dimensional single particle functions following equation 7 but substituting Ψ by φ in the left hand side and with a different set of time-dependent coefficients. This gives rise to a set of nested expansions that can be depicted in ML-trees [60] (see Figure S10 and following). The last layer of SPFs is expanded as a sum of time-independent primitive basis functions $\chi_{l}^{\left(k\right)}$, which are a discrete variable representation (DVR) grid.

(8) $$\varphi_{j_{k}}^{\left(k\right)}\left(q_{k},t\right)=\sum_{l=1}^{N_{l}}\ldots \sum_{n=1}^{N_{n}}c_{l\ldots n,j_{k}}^{\left(k\right)}\left(t\right)\;\chi_{l}^{\left(k\right)}\left(q_{k_{l}}\right)\ldots \chi_{n}^{\left(k\right)}\left(q_{k_{n}}\right)$$

This wavefunction ansatz is then used to variationally solve the time-dependant Schrödinger equation using standard integration techniques [61].

The ML-MCTDH dynamics require analytical potentials, which here are provided by the LVC model. Due to its form, an LVC model is not able to describe dynamics far away from the Franck-Condon point, where direct dynamics are needed to construct the potentials. The ML-MCTDH dynamics are thus used to benchmark the initial dynamics. To compare with DD-vMCG the LVC potentials were constructed based on the first point of the database, i.e., based on the initial vertical energies, ground state frequencies, interstate couplings and state gradients. In this case, the parametrisation was therefore done at the same level of theory as the direct dynamics: TDA-wB97X-D3/cc-pVDZ. To observe the effect of the other solvents, we parametrised a Hamiltonian based on TDA-wB97X-D3/pt(SS+LR)-CPCM/cc-pVDZ level of theory. The number of nuclear degrees of freedom simulated was 57 in the case of the gas phase molecule, 75 in the micro-solvated cluster with water, 93 in the micro-solvated cluster with acetonitrile and 135 with tetrahydrofuran. The ML tree was generated grouping the nine most important modes in higher layers of the tree and distributing the leftover modes in subsequent layers. The importance was decided based on the values of frequency-scaled gradients and couplings along those modes. The ML-MCTDH and MCTDH calculations shown are fully converged with respect to the size of the nuclear basis for the 50 fs simulated. We decided not to propagate the dynamics on the LVC model for longer times, since we believe that the on-the-fly DD-vMCG dynamics should give a better description of the true potential energy surfaces visited.

More details of the simulation parameters, including the number of basis functions and the ML-MCTDH tree are included in the Supplementary Information and in the files available at the UCL repository.

## 3. Results

### 3.1. Analysis of the Experimental Spectra

**Figure 1:**
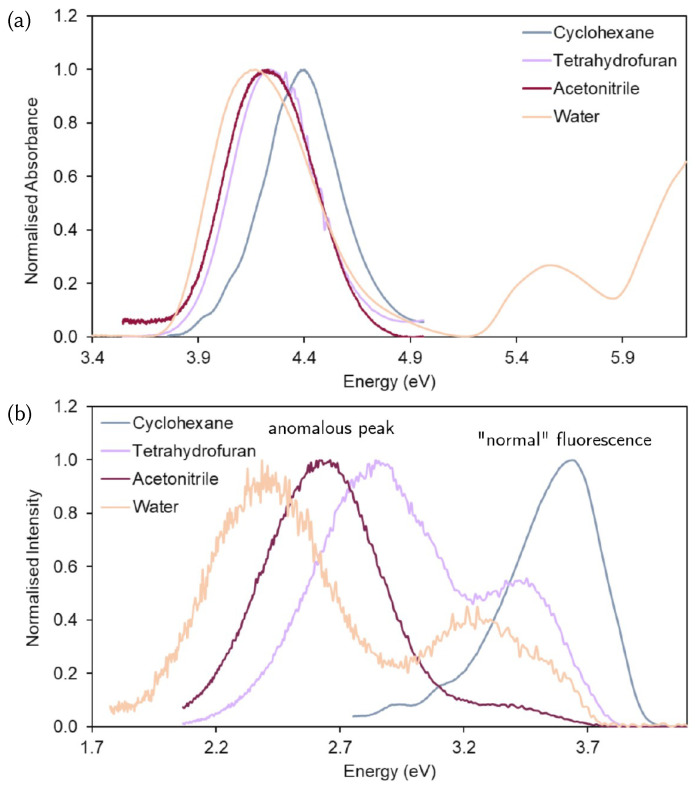
(**a**) Absorption and (**b**) emission spectra of DMABN solutions in cyclohexane, tetrahydrofuran, acetonitrile and water measured with a Perkin Elmer UV/VIS Lambda 365 spectrophotometer and a Horiba Jobin-Yvon Fluorolog-3 spectrofluorometer, respectively.

The absorption spectrum (Figure 1a) shows a Gaussian-like bright band, centred at 4.2–4.4 eV. In cyclohexane this band is blue-shifted with respect to the other solvents. The lack of vibrational fine structure in cyclohexane indicates that there is a high density of vibrational states in the excited electronic state, rather than a broadening due to interactions with the solvent. In the dual-fluorescence spectra, in Figure 1b, we observe a larger shift in the state energies depending on the solvent. The emission peak labelled as “normal” disappears progressively going from cyclohexene to tetrahydrofuran, water and acetonitrile, i.e., with solvent polarity. The “anomalous” dual fluorescence peak is clearly present in water, acetonitrile and tetrahydrofuran, with the transition most red-shifted in water. The spectral noise that can be observed, which should not be confused with a vibrational progression, indicates that the anomalous emission transition is from a dark state. Another reason could be the poor solubility of DMABN in these three solvents, particularly in water.

### 3.2. Analysis of the Potential Energy Surfaces

As a first step, the excited states of interest present in the Frank-Condon region were computed and characterised. While in the following we use TDDFT as a general method, CAS orbitals and energies can be found for comparison in the Supplementary Materials (Figure S1 and Table S2), as well as torsional scans comparing CASPT2 with EOM-CCSD and TDDFT (Figure S2).

In addition to the ground-state equilibrium structure, GS${}_{\min}$, four relevant points in the potential energy surfaces were examined: the L${}_{b}$ dark state that corresponds to the S${}_{1}$ state at the optimised geometry; the L${}_{a}$ bright state that is the S${}_{2}$ state considering a vertical excitation from the GS${}_{\min}$; the local excited state minimum LE${}_{\min}$ that is an almost planar minimum on the S${}_{1}$ potential energy surface and the torsional intermolecular charge transfer state (ICT or, as we will use from now on, CT) minimum on the S${}_{1}$ surface CT${}_{\min}$.

**Figure 2:**
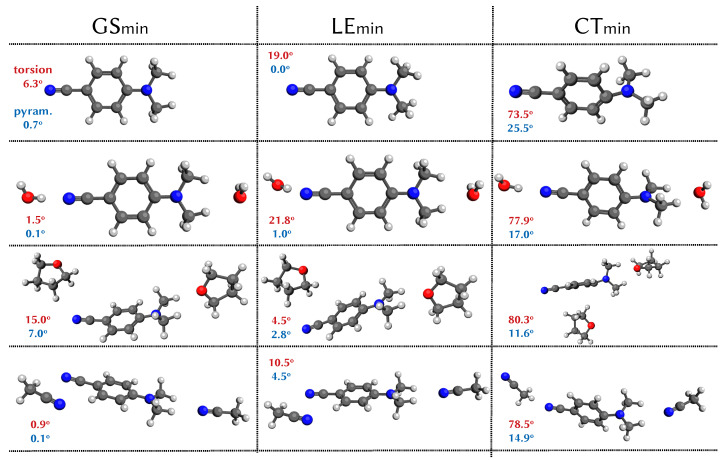
Optimised structures for the ground state (GS), local excited state (LE) and intramolecular charge transfer state (CT) of DMABN in the gas phase and in the micro-solvated clusters (water, acetonitrile and tetrahydrofuran) calculated with TDA-wB97X-D3/LR-CPCM/cc-pVDZ. The value in red for each structure is the torsion of the dimethyl-amino unit relative to the ring, and the value in blue is the pyramidalisation angle of the amino nitrogen relative to the ring plane.

In Figure 2, the optimised geometries of the ground, LE and CT states are shown. The LE minimum structures are very similar to the ground state optimised geometries. We observe more differences among the charge transfer structures, where a different degree of pyramidalisation can be observed, being the largest in the gas phase and water (25.5 and 17.0 degrees, respectively). This degree of pyramidalisation lowers the CT state energy, breaking the approximate C${}_{2v}$ symmetry of the GS${}_{\min}$, as already predicted by the CC2, CCSD and ADC2 methods [28,62].

The TDDFT energies of the gas phase system, listed in Table 2, are in good agreement with CASSCF and EOM-CCSD, but are about 1 eV above the CASPT2 results and the experimental result. This discrepancy can be attributed to the importance of both the dynamic and static correlation in this system, and it is enhanced by the large contribution of double excitations in the CASSCF states (around 20% for S${}_{1}$ and S${}_{2}$). One can observe however, that the potential energy curves along the torsion have a very similar topography regardless of the method used to calculate them, with the S${}_{1}$ and S${}_{2}$ states becoming degenerate at an angle of 45${}^{\circ }$, while S${}_{2}$ and S${}_{3}$ become degenerate at 90${}^{\circ }$. The character of the states is also the same for every level of theory used to calculate the single molecule.

The effect of an environment, represented as two explicit solvent molecules plus PCM, on the TDDFT energies are also listed in Table 2. The energies in cyclohexane are found to be identical to those in the gas phase, with the L${}_{a}$ state approximately 0.2 eV higher in energy. The L${}_{a}$ and L${}_{b}$ states, however, are found to be near-degenerate at the Franck-Condon point in calculations with polar environments. This effect is also observed and even enhanced with CASPT2 including a PCM, with the states swapping order for water and acetonitrile (see Table S2). The switch of CT and LE states in the Frank-Condon region in polar solvents has also been shown by Lunkenheimer et al with high level methods (ADC2/COSMO), confirming the ability of the TDA-wB97X-D3/pt(SS+LR)-CPCM/cc-pVDZ combination to account for this solvent effect. The effect of including the PCM environment on the potential surfaces along the torsion are shown in the Supplementary Materials (Figure S3).

**Figure 3:**
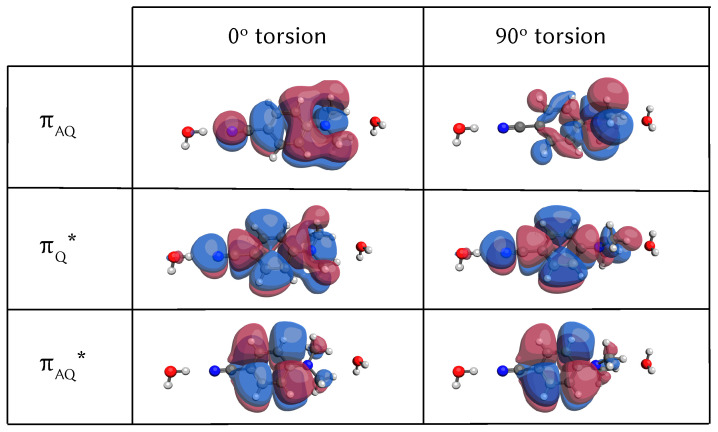
Evolution of the DFT frontier orbitals along the torsion of the NMe2 group extracted from the TDA-wB97X-D3/cc-pVDZ scan that can be found in Figure S3. 0° means optimised GS minimum geometry. The 90° orbitals resemble very closely the ones of the CT minimum (ignoring the pyramidalisation of the carbon next to the NMe2 group) and show clearly that the CT character comes from the hole orbital $\pi_{AQ}$, where the electronic density moves towards the methylene group.

The orbitals from the TDDFT scan along the torsion for the DMABN and two water molecular cluster can be seen in Figure 3. At the optimised geometry (0º column in the figure), the first excited state has a $\pi_{AQ}\pi_{AQ}^{*}$ character and it is labelled as L${}_{b}$, whereas the second excited state L${}_{a}$, the bright state, is generated from the promotion of a $\pi_{AQ}$ electron to a $\pi_{Q}^{*}$ orbital. The “Q” in the orbital tag refers to “quinoline-like” and indicates electronic density on the middle C=C bonds of the ring. The tag “AQ” denotes an antiquinoline orbital, where there is a nodal plane that changes the sign of the electronic wavefunction on the same bonds. Along the torsion, the orbital $\pi_{AQ}$ is the most affected and one can observe a transfer of electronic density from the nitrile group to the dimethylamino end. At this completely torsionated geometry, the lowest excited state has a $\pi_{AQ}\pi_{Q}^{*}$ character that resembles very closely the minimum of the intramolecular charge transfer state (CT). These molecular orbitals look exactly the same as the ones of the single molecule in the gas phase and as the ones from a TDA-wB97X-D3/LR-CPCM/cc-pVDZ calculation. The potential energy scans along the torsion for the water case can be found in Figure S3.

### 3.3. DD-vMCG Excited State Dynamics

**Figure 4:**
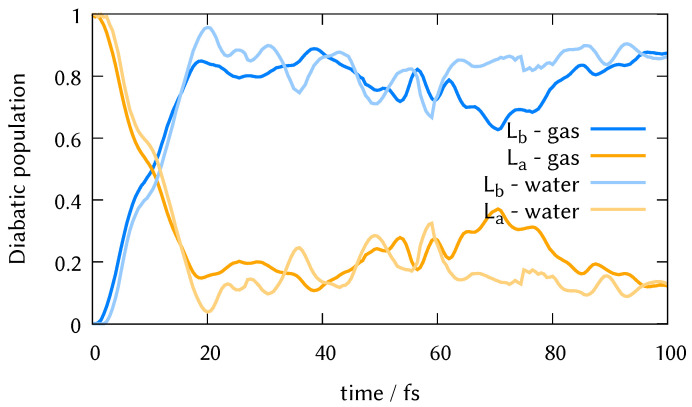
DD-vMCG diabatic electronic populations using 8 nuclear Gaussians starting in the L${}_{a}$ state for the molecule in the gas phase and for the microcluster formed by DMABN and two water molecules.

We performed on-the-fly quantum dynamics using the DD-vMCG method in the gas phase molecule and the micro-solvated system formed by DMABN and two water molecules. The dynamics started in the L${}_{a}$ (S${}_{2}$ state in the Frank-Condon region) to reproduce the experiment by Fuß et al. performed in 2002 [63]. The results using 8 Gaussian wave packets can be seen in Figure 4. Our calculations show clearly that the L${}_{a}$ state is photostable and after one femtosecond undergoes ultrafast internal conversion to the L${}_{b}$ state that is completed in 20 fs, after which there is a population exchange between both states that oscillates in a proportion 80:20. The pronounced recurrence that happens around 70 fs in the gas phase is absent in the micro-solvated dynamics, which is not surprising given the larger phase space of the latter.

A simple exponential decay fit to the state populations (ignoring the initial one femtosecond plateau) with the shape

(9) $$P_{L_{a}}\left(t\right)=\left(1-a\right)*\exp\left(-\kappa t\right)+a,$$

gives as a rate constant 0.151 ± 0.062 fs${}^{-1}$, that corresponds to a mean lifetime of 1 fs + 6.65 fs = 7.65 fs. This result is in good agreement with [63] that predicted a lifetime of 5 ± 5 fs.

During these ultrafast dynamics of up to one hundred femtoseconds, no torsional motion was observed, and we conclude that the timing is too short to simulate the transfer to the ICT state, in agreement with [36,64].

**Figure 5:**
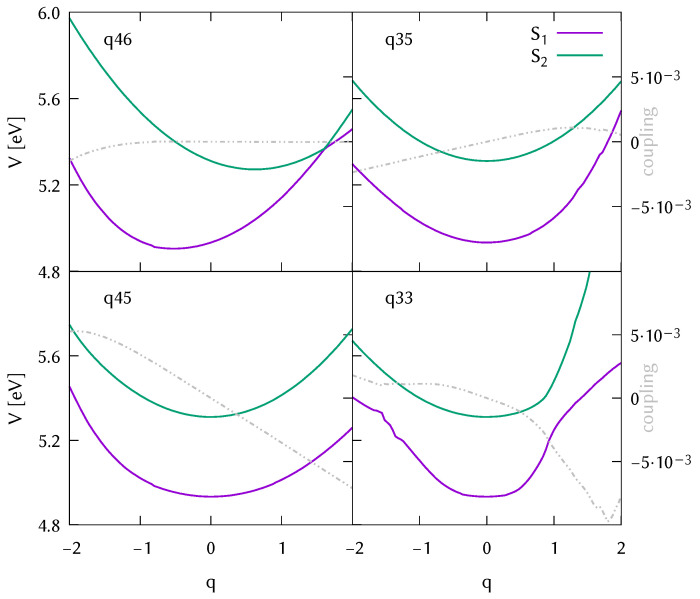
Potential energy surfaces along the four most important normal modes: symmetric C=C stretching and three N-Me${}_{2}$ tilting modes plotted from the database of 601 ab initio points resulting from the TDA-wB97X-D3/cc-pVDZ DD-vMCG dynamics in the gas phase started from the state L${}_{a}$ (S${}_{2}$). The diabatic coupling along those modes is plotted in grey for comparison.

**Figure 6:**
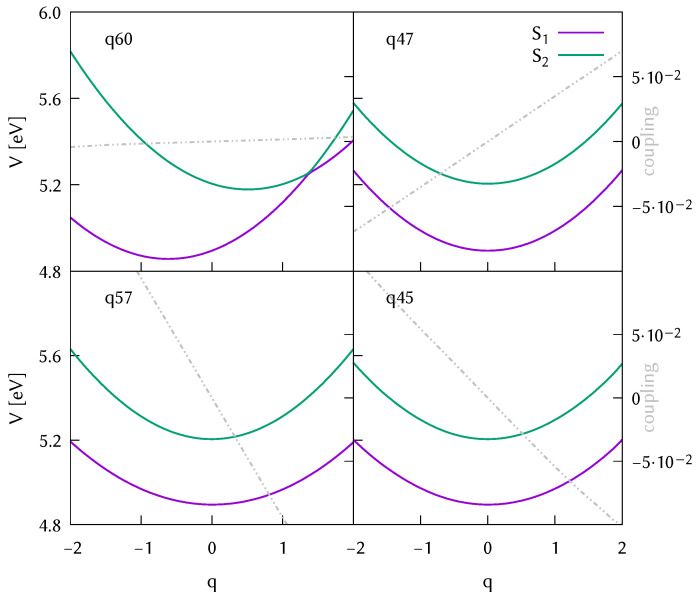
Potential energy surfaces along the four most important normal modes: symmetric C=C stretching and three N-Me${}_{2}$ tilting modes plotted from the database of 220 ab initio points resulting from the TDA-wB97X-D3/cc-pVDZ DD-vMCG dynamics in the water cluster started from the state L${}_{a}$ (S${}_{2}$). The diabatic coupling along those modes is plotted in grey for comparison.

In Figure 5 and Figure 6, we show the adiabatic potential energy cuts from the S${}_{1}$ and S${}_{2}$ states (L${}_{b}$ and L${}_{a}$, respectively in the Frank-Condon region where q=0) for the four modes that drive the dynamics, selected during the analysis of the potential surfaces used to set up model Hamiltonians in Section 3.4. The potential cuts shown are extracted from the databases of ab initio points. The cuts are generated using the Shepard interpolation, as in the dynamics. The databases were composed of 601 different geometries (with their energies, gradients, frequencies, couplings) for the isolated molecule, and 220 geometries for the water-DMABN cluster. The difference in the size of the database indicates that a larger configuration space was visited in the case of the gas phase simulations.

Comparing the potential energy cuts in the gas phase and water, we can observe a shift of the conical intersection along the symmetric C=C stretching that would make it more accessible in the case of the water cluster. The diabatic couplings shown in dashed grey are about one order of magnitude larger in the water solvated system, although it seems to have little effect in the population transfer. In the lower left panel, for mode q33/q45 we observe a larger difference. For the micro-solvated system, the potential is purely harmonic, whereas for the isolated molecule we see hints of a double conical intersection. In any case, the expectation value of the displacement along this normal mode reaches a maximum value of −0.2 after 20 fs (see Figure S8) and therefore it seems that the behaviour at −1 and 1 displacement units does not affect the initial population transfer.

### 3.4. Normal Mode Analysis of Dynamics

**Figure 7:**
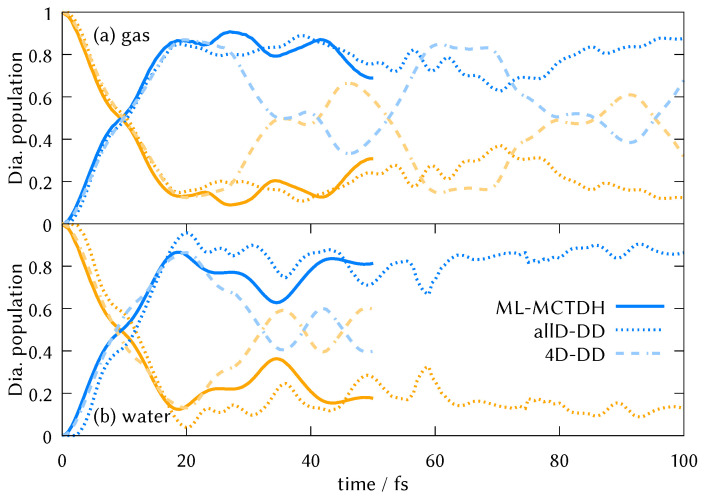
Comparison of the (**a**) gas phase and (**b**) water cluster time evolution of the diabatic populations when running direct dynamics in full dimensionality (dotted line), in four dimensions (dashed line) corresponding to the most important normal modes (see Figure S4) and in the parametrised LVC potentials in full dimensionality with the ML-MCTDH method (solid line).

To determine which molecular movements are responsible for the L${}_{a}$ L${}_{b}$ population transfer, we parametrised linear vibronic coupling Hamiltonians for DMABN in both the gas phase and in water, based on the information of the first point created in the database where we calculated energies, gradients and Hessians from each electronic state and nonadiabatic coupling for each pair of states. On these model potentials we ran full dimensional ML-MCTDH dynamics [61] and observed that the initial population transfer in the dynamics started from the L${}_{a}$ state is very well reproduced (Figure 7). Ordering the normal modes by their λ and κ parameter values, we identified four normal modes that are the main vibrations responsible for this initial population transfer: the C=C ring stretching mode and three modes that involve tilting of the N-dimethyl group. The normal mode vectors can be found in the Supplementary Materials (Figure S4). These modes resemble the coordinates of the branching space of the conical intersection found by the group of Robb [27] with the exception that they saw some pyramidalisation and we do not see any out-of-plane motion during the initial dynamics.

For the water microcluster, we arrived at the same conclusions and these four normal modes also dominate the early-time dynamics. The tuning mode, that carries the gradient in the Frank-Condon region (q46 in the gas phase case and q60 in the water cluster), is the symmetric C=C stretch of the benzene ring, that is the motion that strongly affects the quinoline and antiquinoline-like orbitals (see Figure 3). As can be seen in Figure 5, top left panel, the L${}_{a}$ and the L${}_{b}$ state have their minima at opposite displacements of this normal mode coordinate, since elongated C=C distances would stabilise the $\pi_{AQ}$ and the $\pi_{AQ}^{*}$ orbitals but destabilise the quinoline type orbitals $\pi_{Q}^{*}$ due to the localisation of the electronic density over those C=C bonds. This motion had already been identified by Robb, Curchod and Kochman as the main motion [24,33,36].

To confirm the key role played by these four modes, DD-vMCG calculations were then performed freezing every normal mode except these four to calculate the decay dynamics of the L${}_{a}$ state. These are also shown in Figure 7. DD-vMCG dynamics freezing every mode except the stretching and the three tilting modes (4D-DD) can predict the initial decay of DMABN in the gas phase and the water cluster. This result is also in very good agreement with full dimensional ML-MCTDH dynamics calculations in a parametrised LVC model, indicating that harmonic oscillators in four dimensions and two electronic states is enough to describe the initial ultrafast internal conversion from the L${}_{a}$ state. After 25 fs, however, the 4D dynamics diverge from the full-mode dynamics, especially in the case of the isolated DMABN molecule in the gas phase. An identical result can also be observed in 4D-MCTDH dynamics in Figure S9. This possibly indicates the existence of other conical intersections involving other degrees of freedom and resembling the Tully model II, as anticipated recently by Ibele and Curchod [65].

**Figure 8:**
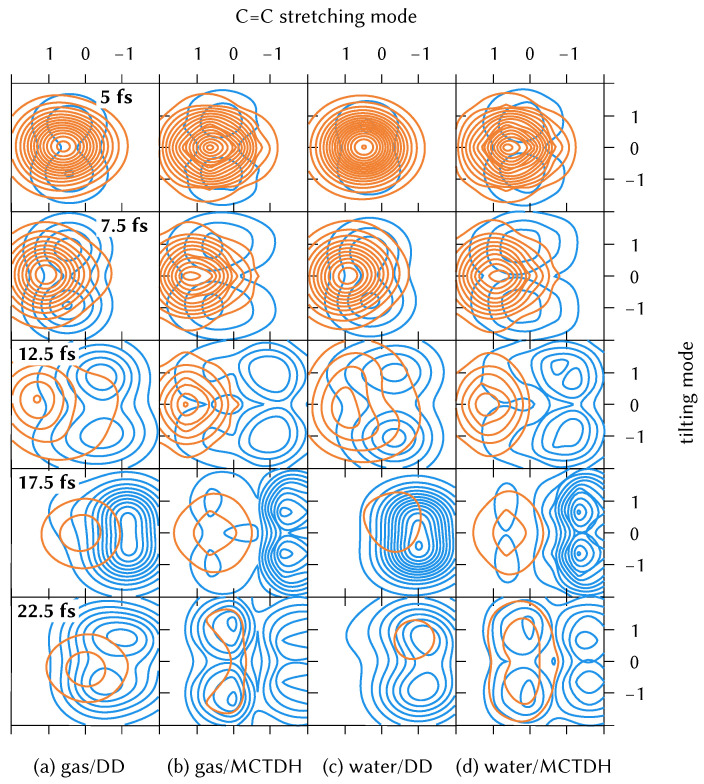
Comparison of the time evolution of the diabatic reduced density (orange on the L${}_{a}$ state and blue on the L${}_{b}$) along the C=C stretching (q46 for the gas phase case and q60 for the water cluster) and the tilting mode that shows the largest coupling (q45 and q57, respectively) for the 4-mode DD-vMCG and MCTDH dynamics on the LVC potentials.

To examine the behaviour of the wavepacket in the early-time dynamics, the reduced density on the L${}_{a}$ and L${}_{b}$ states in the plane of the C=C stretching mode and the main tilting mode (top and bottom left panels of Figure 5 and Figure 6, respectively) is plotted in Figure 8. These are taken from the full dimensional DD-vMCG calculations and, for ease of plotting, a 4-mode MCTDH calculation. After 25 fs, the 4 mode calculations differ from the all-mode ones, and therefore the evolution of the wavepacket is not shown after this time.

Each column of Figure 8 presents a different propagation. It is noticeable how well a superposition of only 8 Gaussians in DD-vMCG (columns 1 and 3) follows the exact wavepacket from the fully converged MCTDH simulations (columns 2 and 4) as it passes through the conical intersection. The large coupling along the tilting coordinate produces a symmetric splitting of the wavepacket that is present as soon as the population is transferred to the L${}_{b}$ state (in blue in the figure) until the 12.5 fs frame. After this time we can observe larger differences between DD-vMCG and MCTDH dynamics: the MCTDH wavepacket splits in both degrees of freedom and delocalises among the regions shown in the plot, whereas the DD-vMCG density prefers to be localised at negative positions of the stretching normal mode. We cannot conclude if this effect is a product of the limitation of the LVC potentials, or a consequence of the small nuclear basis in the direct dynamics calculations.

It is clear in Figure 8 that at t = 0 the wavepacket tail is already spanning the intersection seam, which is why the population transfer is very efficient. This is a challenge for dynamics methods that do not start the dynamics as the exact eigenstate of the ground state harmonic oscillator. In these methods, the initial basis functions (or trajectories) may require motion towards the intersection, to compensate for the lack of density in this region. This situation also makes it difficult to compare simulations that start in the diabatic picture with those starting in the adiabatic, as the initial wavefunctions are quite different.

### 3.5. Solvent Effects: ML-MCTDH Dynamics on Parametrised Hamiltonians

In order to unravel the L${}_{a}$ decay dynamics using a more complete description of the solvent, we parametrised LVC potential energy surfaces including a PCM in addition to the two solvent molecules of the micro-solvated clusters investigated above. The level of theory used was TDA-TDDFT with the wB97X-D3 functional in Q-Chem and C-PCM using the corrections of ptSS and ptLR for the excitation energies. The vertical energies can be found in the last column of Table 2 and places the L${}_{a}$ and L${}_{b}$ states at the same energy.

**Figure 9:**
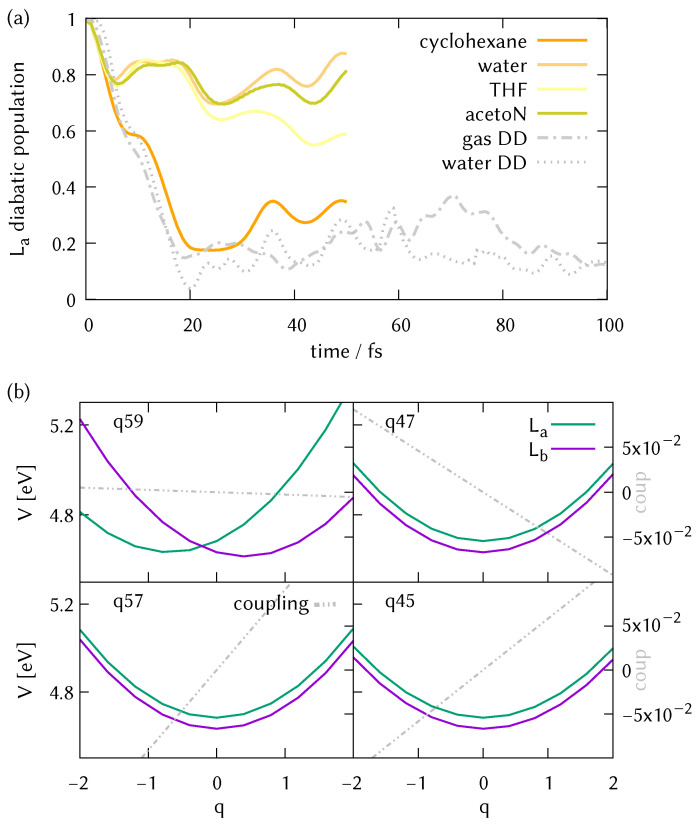
(**a**) Fully converged ML-MCTDH diabatic electronic populations of DMABN in solvent from dynamics starting in the L*a* state. Based on the pt(SS+LR)-PCM/wB97X-D3/cc-pVDZ level of theory and including two explicit solvent molecules except for the cyclohexane case. The light grey lines are the DD-vMCG results for the gas phase and micro-solvated DMABN in water for comparison. (**b**) Diabatic potential energy surfaces and diabatic couplings along the normal modes previously defined as most important for the water micro-solvated system described at the TDAwB97X-D3/pt(SS+LR)-CPCM/cc-pVDZ level of theory.

The state population dynamics of the first 50 fs in LVC models of all 4 solvents, calculated using ML-MCTDH, are show in Figure 9a, with the results from the DD-vMCG simulations of the micro-solvated DMABN in water (no PCM) and the gas phase also shown as a comparison. Using LVC for cyclohexane (where no explicit solvent molecules have been included in the simulation), the decay resembles the one observed by direct dynamics in the gas phase and micro-solvated water. Interestingly, instead of enhancing the decay as might be expected, the new dynamics in every polar solvent shows reflection back to the L${}_{a}$ state after 7 fs. This can be related to the significant effect the PCM has on the potential surfaces, with the L${}_{a}$ and L${}_{b}$ states now being degenerate, as shown in the potential energy surfaces in Figure 9b and in the cut along the torsion in Figure S3 for the water micro-solvated system. The PCM environment causes a strong vertical shift in the potential surfaces, moving the intersection closer to the Franck-Condon point. Approximate adiabatic populations of the initial diabatic wavepacket correspond to a superposition of states in a ratio S${}_{1}$/S${}_{2}$ of 60:40 (based on an MCTDH calculation of a reduced system on the same LVC potentials). The resulting diabatic relaxation is significantly slower.

To benchmark the dynamics, we had planned on using the largest level of theory used to calculate the electronic state energies and characters, SA(4)-MS-CAS(12,11)PT2/cc-pVDZ, to construct a new linear vibronic coupling Hamiltonian, calculating the gradients and state couplings using wavefunction overlaps along the normal mode displacements [66]. However, the active space was not stable for many displaced geometries. Intruder states were observed, and there were unbalanced reference weights of the CASSCF wavefunction. For this reason, the active space was reduced to 6 electrons in 5 orbitals (marked with a pink star in Figure S1), obtaining very similar excitation energies to the CAS(11,12) case (Table S2). On this LVC PES, an ML-MCTDH simulation was run starting in the L${}_{a}$ state. A much slower decay was observed than with TDDFT (black dashed line in Figure 9a). In fact, the first few femtoseconds seem to share the same decay, but there is a competing process that slows down the transfer to the L${}_{b}$ state. The general decay seems to agree with the TDDFT result parametrised for the THF solvation, although better potentials that are parametrised considering the phase space outside the Frank-Condon region, would be desirable to obtain more reliable results.

The decay rates we obtain in our direct dynamics simulations are very similar to those in [35,36,65], in which TDDFT was also used for the electronic structure. A very similar decay rate is also observed in ADC2 in the gas phase, water and acetonitrile in [33,34,64]. The authors concluded that the solvent environment does not affect the initial internal conversion. In our work, however, this conclusion seems to be dependant on the level of theory and description of the environment used.

## 4. Discussion

In this work we have used spectroscopic techniques, as well as a range of electronic methods coupled with quantum dynamics simulations to study the energetics of DMABN and see how the solvent environment affects its early-time dynamics after excitation to the L${}_{a}$ state. All calculations show that, in agreement with earlier work, in the first 20 fs, the system passes through a conical intersection irrespective of the environment.

As a test of our dynamics methods, we performed on-the-fly DD-vMCG quantum dynamics on DMABN and the water micro-solvated cluster with TDDFT potentials and compared them to fully converged MCTDH and ML-MCTDH quantum dynamics on simple parametrised LVC potentials at the same level of theory. As this is the first time that DD-vMCG has been used to treat such large systems (57 and 75 degrees of freedom for the single molecule and the micro-solvated cluster), it stands out, how well a vMCG wavepacket follows the exact wavepacket, even with a small number of Gaussians in the nuclear expansion. This demonstrates that DD-vMCG is a powerful tool for accurately describing the passage through a conical intersection including all phase effects.

Linear vibronic coupling models have been proved to be very useful for this system and constitute a simple method to benchmark the initial dynamics, but it should be used carefully for longer times as the harmonic nature of the model does not allow long-range motions to occur. Thus, potential energy surfaces calculated on the fly will diverge and provide the flexibility needed to bring the nuclear basis functions to places that are inaccessible to harmonic potentials.

From an analysis of the dynamics, we have identified the key motions driving the ultrafast deactivation from the L${}_{a}$ state as the quinoline C=C stretching and three similar NMe${}_{2}$ tilting modes. No out-of-plane motions were found to play a role in the early dynamics. The same modes were involved in both the gas and micro-solvated systems.

Finally, LVC models and quantum dynamics simulations were used to examine the effect of the level of theory and description of the environment on the dynamics. Through improving the description of polar solvents by using C-PCM with the pt(SS+LR) corrections to the energy, after an initial decay for the micro-solvated system, the wavepacket is reflected back to the L${}_{a}$ state and does not cross to L${}_{b}$. Examination of the state energies and potential surfaces show that this is due to the states becoming degenerate at the Franck-Condon point when the PCM is included. More work is needed to validate these initial results as they indicate a complete change in initial dynamics due to the solvent environment.

More work is also needed to evaluate the need of multireference methods to describe the DMABN system. CASPT2 provides energies much closer to the experimental values, and it strongly affects the population decay: CASPT2 predicts a slower decay from the L${}_{a}$ state when compared to the direct dynamics using TDDFT (about five times slower).

Since the time frame of our quantum dynamic simulations is too short to observe any conversion to the charge transfer state, no remarks can be made regarding the nature of the double emission band in DMABN in the different solvents. The experimental absorption and emission spectra, however, have been used to benchmark the calculated vertical energies of the ground state and excited state optimised structures in several micro-solvated environments.

The work presented here shows that, despite the many years of study, DMABN is still a challenging molecule for theoretical calculations. The photo-excited dynamics is dictated by a balance of coupled electronic states that are clearly sensitive to the environment and electronic structure method used. The combination of DD-vMCG, along with the use of LVC models with full quantum dynamics as a benchmark for early times, is a useful tool for the study of the dynamics, able to capture the nonadiabatic effects accurately, and so provide rigorous tests of the potential surfaces.

## Figures and Tables

**Table 1:** Dielectric constants and refraction indices of the solvents used in this work.

Solvent	Dielectric Constant ϵ	Refraction Index, n
Cyclohexane	2.02	1.43
Tetrahydrofuran	7.58	1.41
Acetonitrile	37.50	1.34
Water	80.10	1.33

**Table 2:** Table showing electronic state character and vertical energies in electronvolts relative to the ground state of the most relevant points of the potential energy surfaces of DMABN in the gas phase and the micro-solvated cluster of DMABN and two water molecules, two tetrahydrofuran molecules and two acetonitrile molecules. The symmetry irreducible representations belong to the C${}_{2v}$ group in the case of GS${}_{\min}$, L${}_{b}$, L${}_{a}$ and LE${}_{\min}$ and to C${}_{s}$ in case of CT${}_{\min}$. ΔE${}_{\exp}$ is the experimental energy measured in this work, ΔE${}_{\mathrm{TDDFT}}$ is the TDA-wB97X-D3/cc-pVDZ calculated energy of the gas phase molecule or the solvent clusters, ΔE${}_{\mathrm{PCM}/\mathrm{TDDFT}}$ is the TDA-wB97X-D3/pt(SS+LR)-CPCM/cc-pVDZ calculated energy of the gas phase molecule or the solvent clusters in a solvent continuum model and f is the oscillator strength of the transition to that state from the ground state.

State Label	Character (symm.)	Solvent	ΔE${}_{\exp}$	ΔE${}_{\mathrm{TDDFT}}$ (f)	ΔE${}_{\mathrm{PCM}/\mathrm{TDDFT}}$ (f)
GS${}_{\min}$	$\pi_{\mathrm{AQ}}^{2}$ (A${}_{1}$)	gas/			
L${}_{b}$	$\pi_{\mathrm{AQ}}\pi_{\mathrm{AQ}}^{*}$ (B${}_{2}$)	cyclohexane		4.82 (0.00)	4.84 (0.03)
L${}_{a}$	$\pi_{\mathrm{AQ}}\pi_{Q}^{*}$ (A${}_{1}$)		4.40	5.08 (0.54)	5.05 (0.68)
LE${}_{\min}$	$\pi_{\mathrm{AQ}}\pi_{\mathrm{AQ}}^{*}$ (B${}_{2}$)		3.90	4.46 (0.04)	4.24 (0.18)
CT${}_{\min}$	$\pi_{\mathrm{AQ}}\pi_{Q}^{*}$ (A”)		3.64	3.05 (0.00)	3.06 (0.00)
GS${}_{\min}$	$\pi_{\mathrm{AQ}}^{2}$ (A${}_{1}$)	water			
L${}_{b}$	$\pi_{\mathrm{AQ}}\pi_{\mathrm{AQ}}^{*}$ (B${}_{2}$)			4.90 (0.04)	4.68 (0.04)
L${}_{a}$	$\pi_{\mathrm{AQ}}\pi_{Q}^{*}$ (A${}_{1}$)		4.17	5.20 (0.76)	4.63 (0.81)
LE${}_{\min}$	$\pi_{\mathrm{AQ}}\pi_{\mathrm{AQ}}^{*}$ (B${}_{2}$)		3.25	4.75 (0.75)	4.28 (1.19)
CT${}_{\min}$	$\pi_{\mathrm{AQ}}\pi_{Q}^{*}$ (A”)		2.38	3.08 (0.00)	2.95 (0.00)
GS${}_{\min}$	$\pi_{\mathrm{AQ}}^{2}$ (A${}_{1}$)	THF			
L${}_{b}$	$\pi_{\mathrm{AQ}}\pi_{\mathrm{AQ}}^{*}$ (B${}_{2}$)			4.87 (0.03)	4.70 (0.03)
L${}_{a}$	$\pi_{\mathrm{AQ}}\pi_{Q}^{*}$ (A${}_{1}$)		4.24	5.18 (0.73)	4.69 (0.76)
LE${}_{\min}$	$\pi_{\mathrm{AQ}}\pi_{\mathrm{AQ}}^{*}$ (B${}_{2}$)		3.47	4.81 (0.15)	4.43 (1.11)
CT${}_{\min}$	$\pi_{\mathrm{AQ}}\pi_{Q}^{*}$ (B${}_{2}$)		2.84	3.16 (0.00)	3.09 (0.00)
GS${}_{\min}$	$\pi_{\mathrm{AQ}}^{2}$ (A${}_{1}$)	acetonitrile			
L${}_{b}$	$\pi_{\mathrm{AQ}}\pi_{\mathrm{AQ}}^{*}$ (B${}_{2}$)			4.89 (0.05)	4.68 (0.04)
L${}_{a}$	$\pi_{\mathrm{AQ}}\pi_{Q}^{*}$ (A${}_{1}$)		4.23	5.16 (0.74)	4.66 (0.79)
LE${}_{\min}$	$\pi_{\mathrm{AQ}}\pi_{\mathrm{AQ}}^{*}$ (B${}_{2}$)		3.38	4.83 (0.37)	4.76 (0.91)
CT${}_{\min}$	$\pi_{\mathrm{AQ}}\pi_{Q}^{*}$ (A”)		2.66	3.11 (0.00)	3.05 (0.00)

## Data Availability

The data from the simulations, including the electronic wavefunctions, frequency calculations and quantics inputs for the ML-MCTDH and DD-vMCG simulations are available on the publicly accessible UCL repository (doi:10.5522/04/17069681). The Quantics program is hosted on Gitlab, and available on request to the corresponding authors.

## Supplementary Materials

The following are available online. Figure S1: RASSCF orbitals used in the active space for the SA(4)-MS-CAS(11,12)PT2/ccpvDZ computations, Figure S2: Potential energy rigid scan of the isolated DMABN molecule along the torsion using CASPT2, EOM-CCSD and TDDFT, Figure S3: Potential energy surfaces of the DMABN molecule with two water molecules along the torsion, Figure S4: Normal mode vectors, Figure S5: Evolution of the L${}_{a}$ state population for different DD-vMCG simulations of the DMABN molecule in gas phase, Figure S6: Evolution of the L${}_{a}$ state population for different DD-vMCG simulations of the cluster of DMABN and two water molecules, Figure S7: Comparison between direct dynamics (DD) and dynamics parametrised with a LVC model, Figure S8: Comparison of the time evolution of the expectation value of the four most important normal modes, Figure S9: Comparison of the (a) gas phase and (b) water cluster time evolution of the diabatic populations when running direct dynamics in full dimensionality (dotted line), in the parametrised LVC potentials with the ML-MCTDH method (solid line) and in MCTDH with the four most important modes (dashed line). Figures S10 and S11: Layer structure of the ML-MCTDH simulations on the potentials parametrised from first database point of the DD-vMCG dynamics of the single DMABN molecule in gas phase (Figure S10), of the cluster formed by one DMABN molecule and two water molecules (Figure S11), Figure S12: Layer structure of the ML-MCTDH simulations on the SA(4)-MSCAS(6,5)PT2/cc-pvDZ parametrised potentials, Figures S13–S16: Layer structure of the ML-MCTDH simulations on the pt(SS+LR)-CPCM/wB97X-D3/cc-pVDZ parametrised potentials applied to the DMABN molecule in a continuum solvation of cyclohexane (Figure S13), of water (Figure S14), of acetonitrile (Figure S15) and of THF (Figure S16), Table S1: Benchmarking of TDDFT/wB97X-D3 with different basis sets and solvent models, Table S2: Benchmarking among different electronic structure methods.

## Abbreviations

The following abbreviations are used in this manuscript:

DMABN	1,1-dimethylaminobenzonitrile
DD-vMCG	Direct dynamics variational multiconfigurational Gaussian
MCTDH	Multiconfigurational Time-dependent Hartree
CASPT2	Complete active space perturbation theory to second order
TDDFT	Time-dependent density functional theory
LVC	Linear Vibronic Coupling
PES	Potential Energy Surface
