# Supplementary material for: Micro-Solvated DMABN: Excited State Quantum Dynamics and Dual Fluorescence Spectra
Source: Molecules. 2021 Nov 29;26(23):7247. doi: 10.3390/molecules26237247 (PMC8658867; doi:10.3390/molecules26237247)
Supplement: Supplementary file 1 [file molecules-26-07247-s001.zip › molecules-1462774-supplementary.pdf]

SuppInfo for: Microsolvated DMABN: excited state DD-vMCG dynamics and double fluorescence spectra

Sandra Gómez, Esra N. Soysal, Graham A. Worth

October 29, 2021

1 Quantum Chemistry benchmark

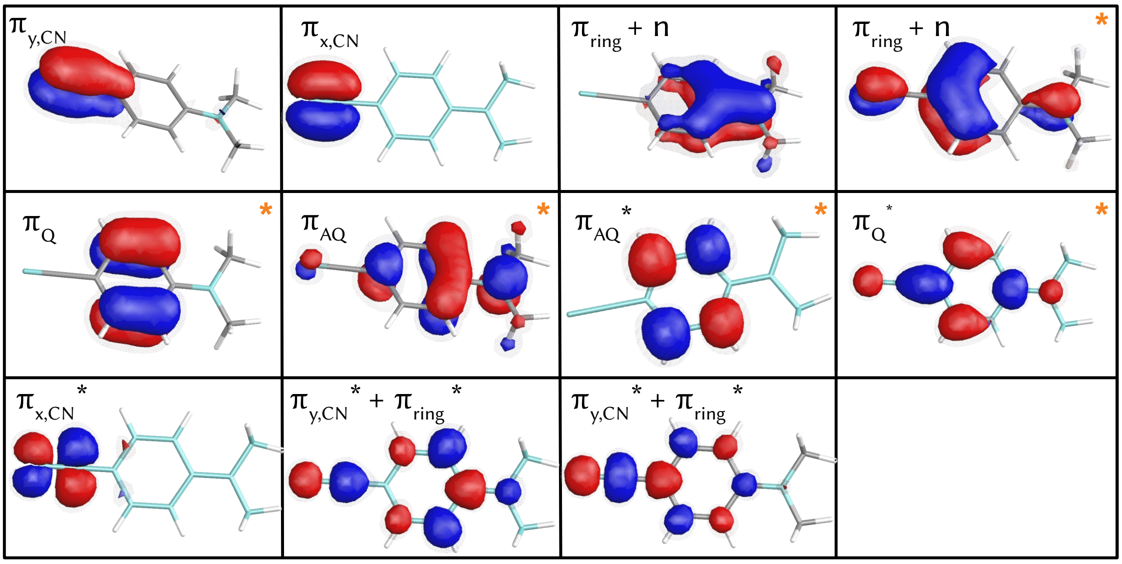

SI-Figure S1: RASSCF orbitals used in the active space for the SA(4)-MS-CAS(11,12)PT2/cc-pvDZ computations. The orange asterisk indicates the smaller active space used for the SA(4)-MS-CAS(6,5)PT2/cc-pvDZ calculations.

SI-Table S1: Benchmarking of TDDFT/wB97X-D3 with different basis sets and solvent models using the LR-C-PCM method and different amount of solvent molecules to simulate the environment.

Solvent	Solvent Method	Basis Set	Lb (eV)	La (eV)	LE (eV)	CT (eV)
Gas		cc-pVDZ	4.86	5.23	4.51	3.02
Water	PCM	cc-pVDZ	4.72	4.78	4.18	3.27
	Two explicit solvent molecules + PCM	cc-pVDZ	4.71 (4.59)	4.71 (4.59)	4.12 (4.00)	3.15 (3.14)
	Two explicit solvent molecules	aug-cc-pVDZ	4.64	4.88	4.34	3.28
Tetrahydrofuran	PCM	cc-pVDZ	4.72	4.79	4.27	3.32
	Two explicit solvent molecules + PCM	cc-pVDZ	4.69 (4.58)	4.73 (4.63)	4.21	2.95
	Two explicit solvent molecules	cc-pVDZ	4.76 (4.64)	4.97 (4.88)		
	One explicit solvent molecule + PCM	cc-pVDZ	4.73 (4.60)	4.77 (4.67)		
	One explicit solvent molecule	cc-pVDZ	4.81 (4.68)	5.05 (4.95)		
Acetonitrile	PCM	cc-pVDZ	4.72	4.78	4.19	3.28
	Two explicit solvent molecules + PCM	cc-pVDZ	4.71 (4.59)	4.74 (4.62)	4.16	3.21
	Two explicit solvent molecules	cc-pVDZ	4.77 (4.63)	4.97 (4.86)		
	One explicit solvent molecule + PCM	cc-pVDZ	4.73 (4.60)	4.75 (4.63)		
	One explicit solvent molecule	cc-pVDZ	4.82 (4.69)	5.03 (4.93)		

SI-Table S2: Benchmarking among different electronic structure methods using the cc-pvDZ basis set. Vertical energies with respect to their ground state shown in electronvolt. Calculations performed at the ground state minimum optimised with MP2/cc-pvdz. The solvent is simulated with SS-PCM as implemented in MOLCAS

Solvent	CAS(6,5)PT2			CAS(11,12)SCF			CAS(11,12)PT2			EOM-CCSD		
	L_b	L_a	S3	L_b	L_a	S3	L_b	L_a	S3	L_b	L_a	S3
Gas	3.57	4.53	5.54	4.56	5.96	7.04	3.89	4.49	5.79	4.59	5.10	6.54
Tetrahydrofuran				5.32	5.68	6.82	3.70	4.50	5.12			
Acetonitrile				4.65	5.54	6.77	3.83	3.53	5.32			
Water				4.65	5.53	6.78	3.83	3.55	5.12			

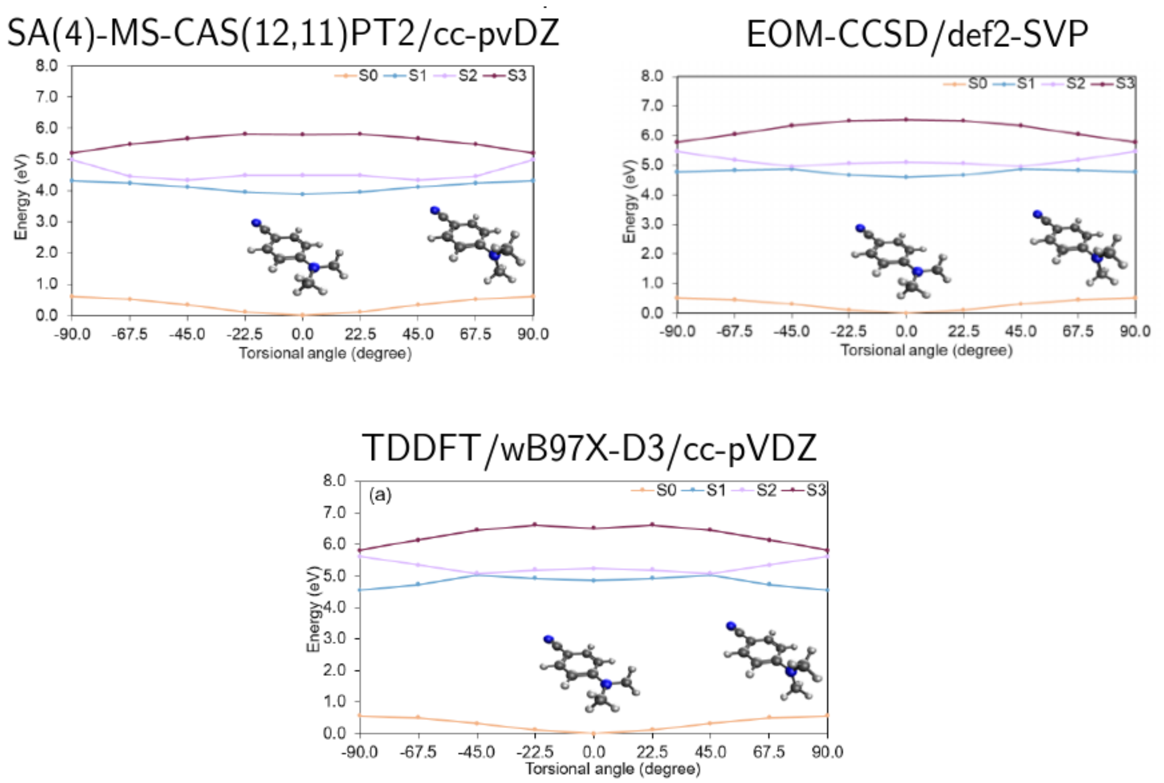

SI-Figure S2: Potential energy rigid scan of the isolated DMABN molecule along the torsion using CASPT2, EOM-CCSD and TDDFT.

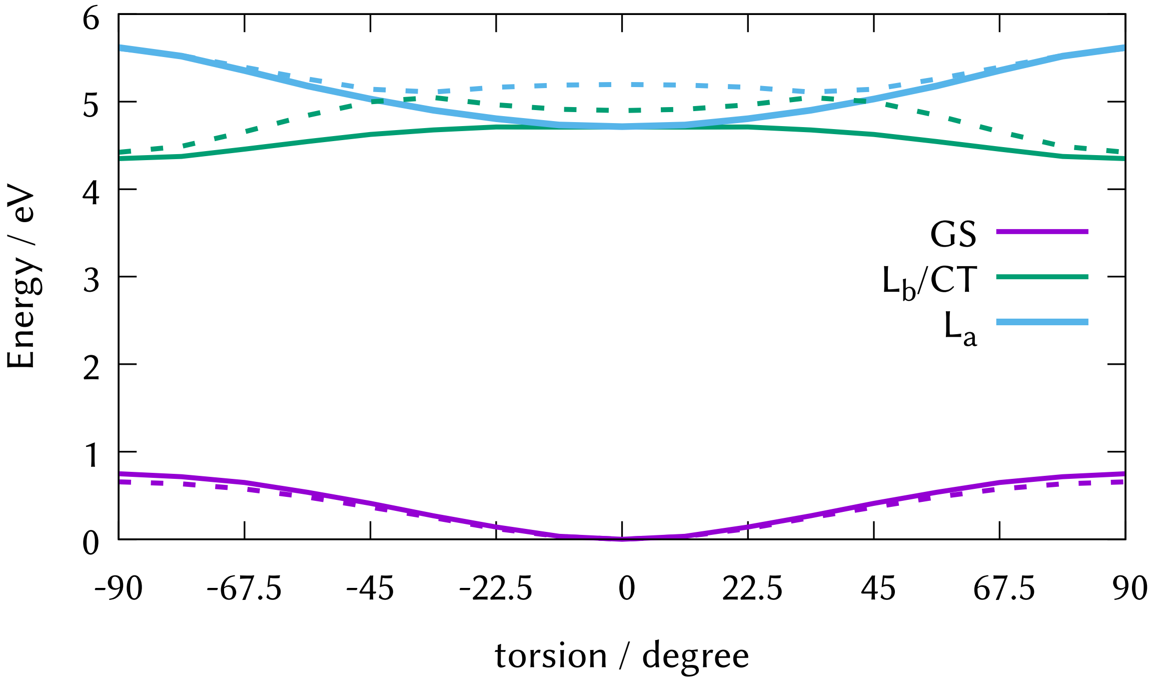

SI-Figure S3: Potential energy surfaces of the DMABN molecule with two water molecules along the torsion using LR-C-PCM/TDDFT/wB97X-D3/cc-pVDZ (solid line) and of the cluster DMABN + two water molecules but without including any PCM solvent effects (dashed).

2 Quantum dynamics analysis

2.1 Most important normal modes

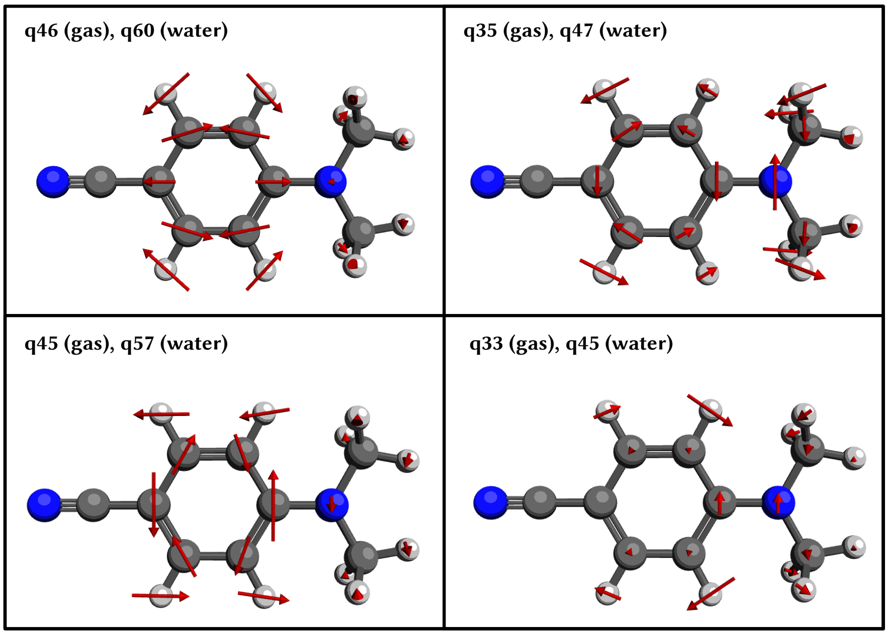

SI-Figure S4: Normal mode vectors (C=C stretching, tilting modes) plotted from the single molecule frequency calculation at the wB97X-D3/cc-pvDZ level of theory. Each panel has a label that denotes the normal mode number to assing them to the potential energy surface cuts and the evolution of the wavepacket for the gas and water direct dynamics calculations and the ML-MCTDH calculations on the surfaces calculated from the first point of the dynamics.

2.2 Convergence with respect to the number of gaussians

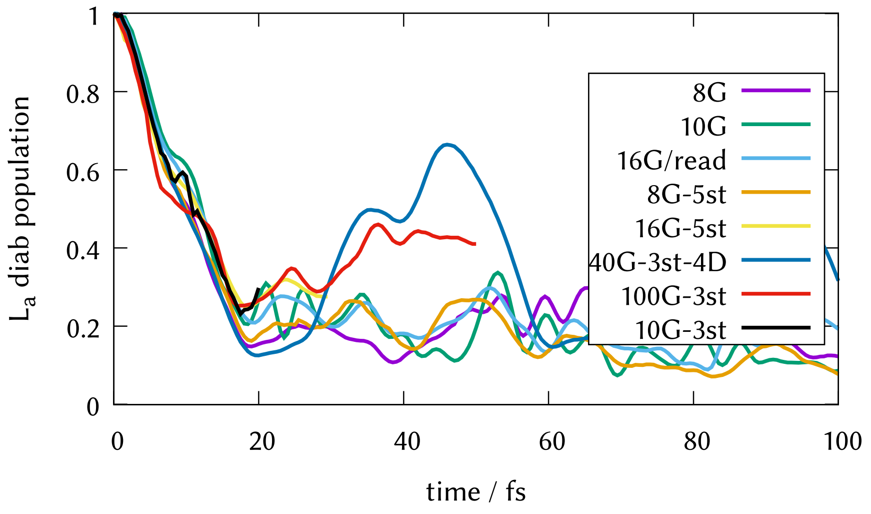

SI-Figure S5: Evolution of the L_a state population for different DD-vMCG simulations of the DMABN molecule in gas phase. 5st and 3st denote the number of states included. 8G,10G, etc, refer to the number of gaussians in the expansion of the nuclear basis and "read" means a calculation on the potentials of a previous simulation, without calculating any new ab-initio points.

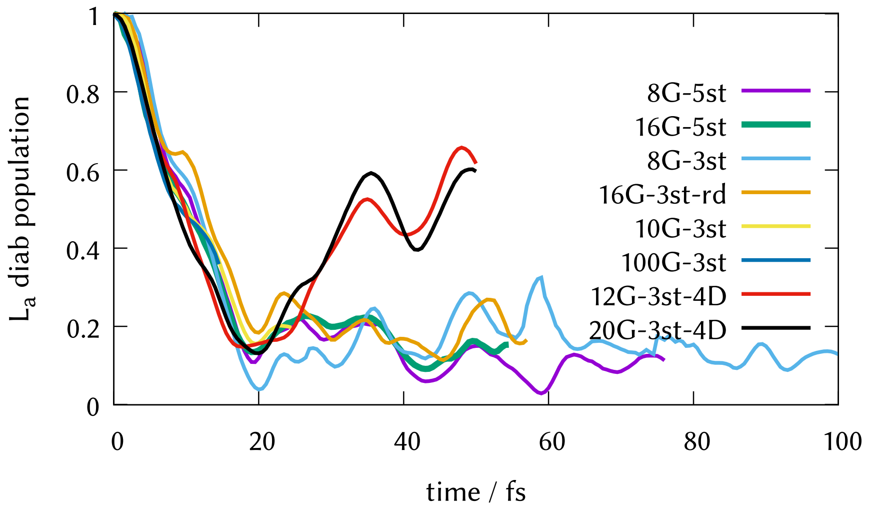

SI-Figure S6: Evolution of the L_a state population for different DD-vMCG simulations of the cluster of DMABN and two water molecules. 5st and 3st denote the number of states included and 8G,10G, etc, refer to the number of gaussians in the expansion of the nuclear basis.

2.3 Dynamics with different subsets of normal modes

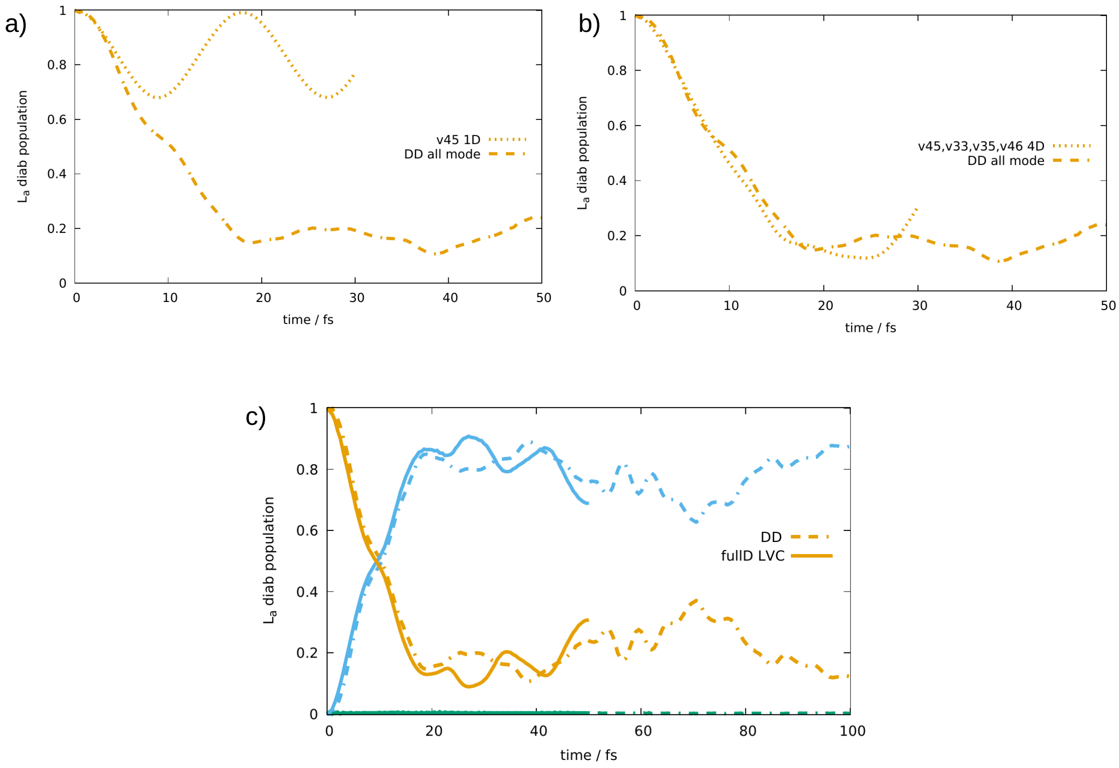

SI-Figure S7: Comparison between direct dynamics (DD) and dynamics parametrised with a LVC model using different subsets of normal modes. a) Population decay of the La state running 1D exact dynamics on the LVC potentials (dotted line) and DD in full dimensionality (dashed). b) Population decay of the La state running 4D MCTDH dynamics on the LVC potentials (dotted line) and DD in full dimensionality (dashed).

c) Population of the La and Lb states running full D ML-MCTDH dynamics on the LVC potentials (continuous line) and DD in full dimensionality (dashed).

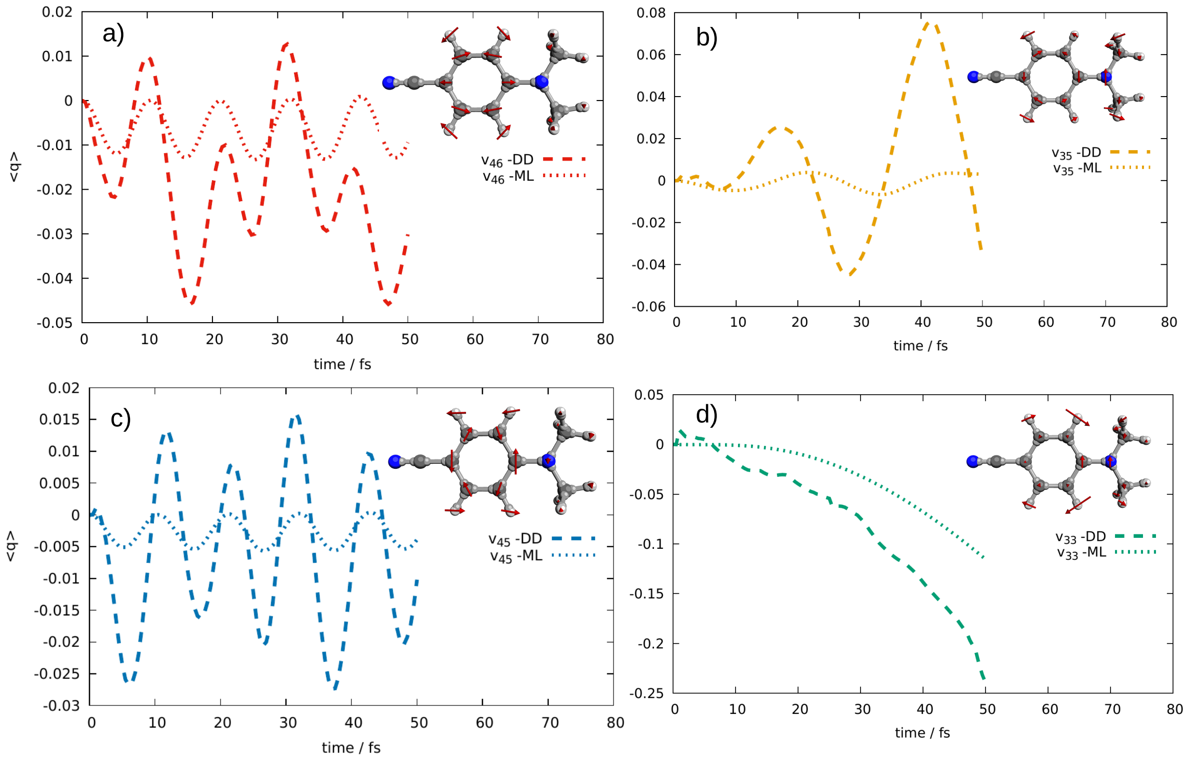

SI-Figure S8: Comparison of the time evolution of the expectation value of the four most important normal modes (symmetric stretch, N-Me_2 wagging,..) for the DD-vMCG dynamics and the ML-MCTDH dynamics on the LVC potentials.

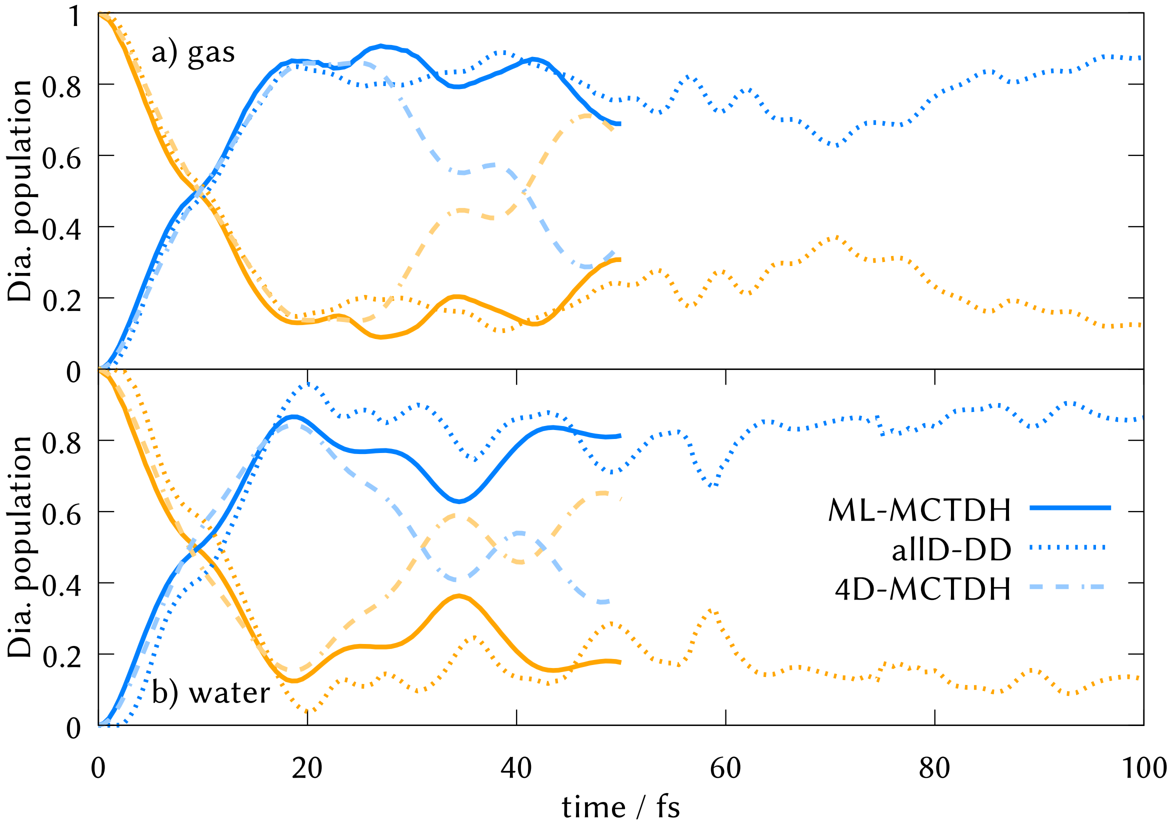

SI-Figure S9: Comparison of the a) gas phase and b) water cluster time evolution of the diabatic populations when running direct dynamics in full dimensionality (dotted line), in the parametrised LVC potentials with the ML-MCTDH method (solid line) and in MCTDH with the four most important modes (dashed line).

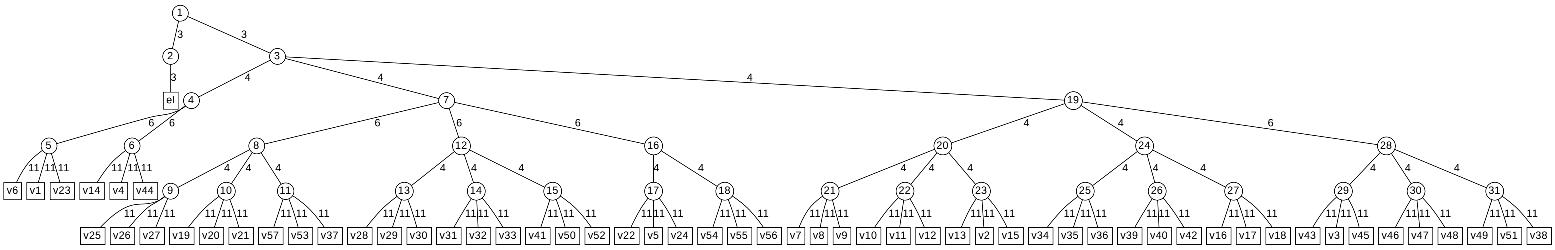

SI-Figure S10: Layer structure of the ML-MCTDH simulations on the potentials parametrised from first database point of the DD-vMCG dynamics of the single DMABN molecule in gas phase.

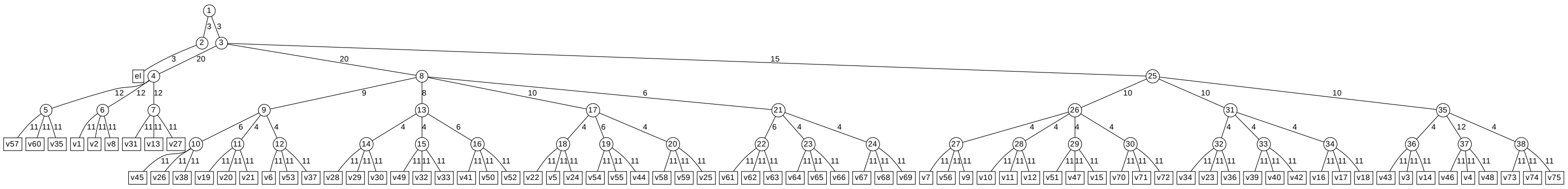

SI-Figure S11: Layer structure of the ML-MCTDH simulations on the potentials parametrised from first database point of the DD-vMCG dynamics of the cluster formed by one DMABN molecule and two water molecules.

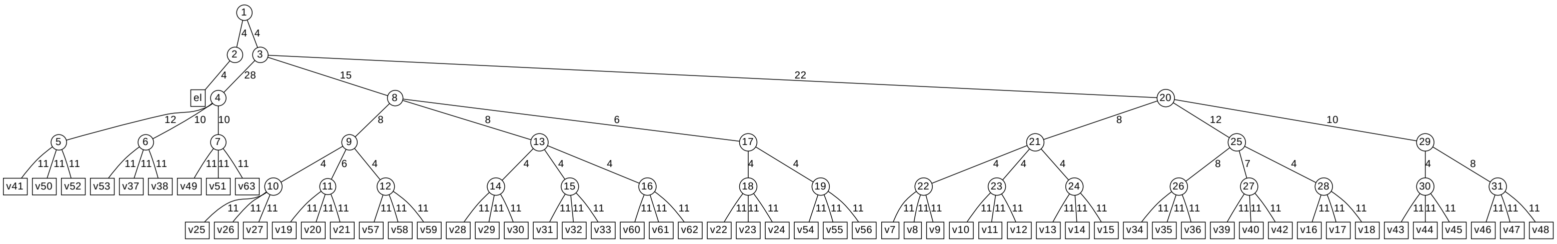

SI-Figure S12: Layer structure of the ML-MCTDH simulations on the SA(4)-MS-CAS(6,5)PT2/cc-pvDZ parametrised potentials.

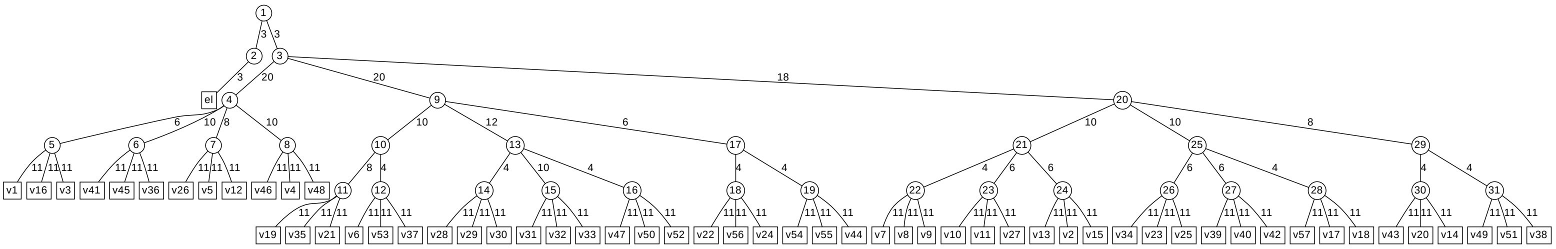

SI-Figure S13: Layer structure of the ML-MCTDH simulations on the pt(SS+LR)-C-PCM/wB97X-D3/cc-pVDZ parametrised potentials applied to the DMABN molecule in a continuum solvation of cyclohexane.

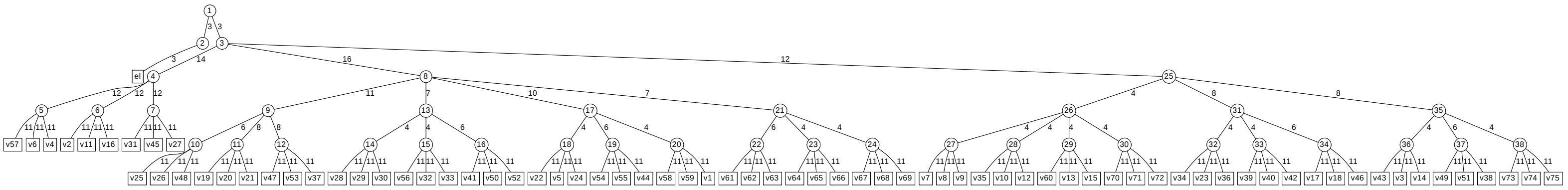

SI-Figure S14: Layer structure of the ML-MCTDH simulations on the pt(SS+LR)-C-PCM/wB97X-D3/cc-pVDZ parametrised potentials applied to the cluster formed by a DMABN molecule and two waters in a continuum solvation of water.

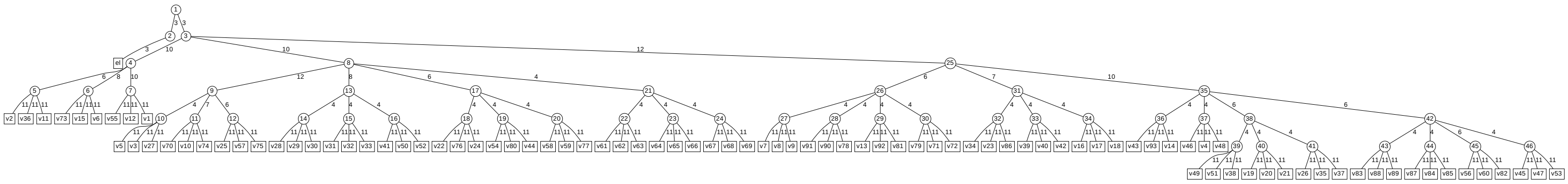

SI-Figure S15: Layer structure of the ML-MCTDH simulations on the pt(SS+LR)-C-PCM/wB97X-D3/cc-pVDZ parametrised potentials applied to the cluster formed by a DMABN molecule and two acetonitrile molecules in a continuum solvation of acetonitrile.

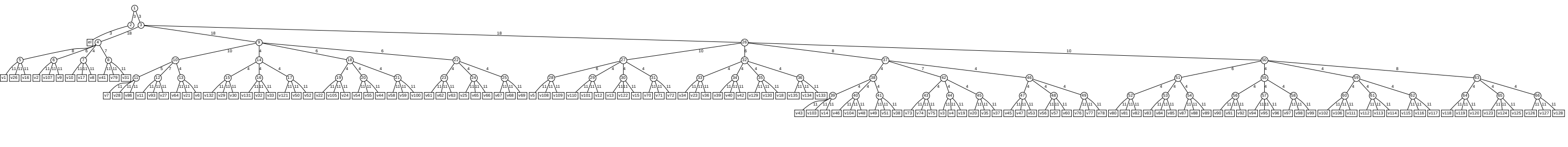

SI-Figure S16: Layer structure of the ML-MCTDH simulations on the pt(SS+LR)-C-PCM/wB97X-D3/cc-pVDZ parametrised potentials applied to the cluster formed by a DMABN molecule and two tetrahydrofurane molecules in a continuum solvation of THF.
